# Correlation of Beta-2 Adrenergic Receptor Expression in Tumor-Free Surgical Margin and at the Invasive Front of Oral Squamous Cell Carcinoma

**DOI:** 10.1155/2016/3531274

**Published:** 2016-03-02

**Authors:** Denise Tostes Oliveira, Diego Mauricio Bravo-Calderón, Gustavo Amaral Lauand, Agnes Assao, José-Manuel Suárez-Peñaranda, Mario Pérez-Sayáns, Abel García-García, Aparecido Nilceu Marana, Suely Nonogaki, José Roberto Pereira Lauris, Luiz Paulo Kowalski

**Affiliations:** ^1^Department of Stomatology, Area of Pathology, Bauru School of Dentistry, University of São Paulo, 17012-901 Bauru, SP, Brazil; ^2^Department of Pathology and Forensic Sciences, University Hospital and School of Medicine of Santiago de Compostela, Santiago de Compostela, 15706 Galicia, Spain; ^3^Faculty of Medicine and Dentistry, Institute of Research of Santiago de Compostela (IDIS), Clinical Hospital University of Santiago, Santiago de Compostela, 15782 A Coruña, Spain; ^4^Department of Computing, Faculty of Sciences, São Paulo State University (UNESP), 17033-360 Bauru, SP, Brazil; ^5^Pathology Division, Adolfo Lutz Institute, 01246-902 São Paulo, SP, Brazil; ^6^Department of Community Dentistry, Bauru School of Dentistry, University of São Paulo, 17012-901 Bauru, SP, Brazil; ^7^Department of Head and Neck Surgery and Otorhinolaryngology, A.C. Camargo Cancer Center, 01509-010 São Paulo, SP, Brazil

## Abstract

*Background*. The beta-2 adrenergic receptor is expressed by neoplastic cells and is correlated with a wide spectrum of tumor cell mechanisms including proliferation, apoptosis, angiogenesis, migration, and metastasis.* Objectives*. The present study aimed to analyze the expression of the beta-2 adrenergic receptor (*β*2-AR) in tumor-free surgical margins of oral squamous cell carcinomas (OSCC) and at the invasive front. Sixty-two patients diagnosed with OSCC, confirmed by biopsy, were selected for the study. The clinicopathological data and clinical follow-up were obtained from medical records and their association with *β*2-AR expression was verified by the chi-square test or Fischer's exact test. To verify the correlation of *β*2-AR expression in tumor-free surgical margins and at the invasive front of OSCCs, Pearson's correlation coefficient test was applied.* Results*. The expression of *β*2-AR presented a statistically significant correlation between the tumor-free surgical margins and the invasive front of OSCC (*r* = 0.383; *p* = 0.002). The immunohistochemical distribution of *β*2-AR at the invasive front of OSCC was also statistically significant associated with alcohol (*p* = 0.038), simultaneous alcohol and tobacco consumption (*p* = 0.010), and T stage (*p* = 0.014).* Conclusions*. The correlation of *β*2-AR expression in OSCC and tumor-free surgical margins suggests a role of this receptor in tumor progression and its expression in normal oral epithelium seems to be constitutive.

## 1. Background

Chronic stress dysregulates the hypothalamic-pituitary-adrenal axis, elevating the production of stress related hormones, epinephrine, and norepinephrine [1]. These catecholamines seem to enhance the expression of vascular endothelial growth factor (VEGF) and the matrix of metalloproteinases (MMPs) in malignant tumors, contributing to tumor progression [2]. It is known that *β*2-AR is a member of a large family of G-protein-coupled receptors and is responsible for transduction signals from catecholamine ligands [3, 4]. Once *β*2-AR binds to catecholamine ligands, it stimulates a G-protein receptor, resulting in adenylyl cyclase activity and, subsequently, in the generation of intracellular cyclic AMP (cAMP) that activates protein kinase A (PKA), which modulates several cellular functions [5]. A growing number of scientific evidences have verified that *β*2-AR is expressed by malignant neoplastic cells and that, under chronic psychological stress, through catecholamine induced activation, it could regulate a wide spectrum of tumor cell mechanisms including proliferation, apoptosis, angiogenesis, migration, and metastases [1, 2, 6–25].

Particularly, investigations in oral cancer cell lines have demonstrated that *β*2-AR signaling upregulates interleukin-6 (IL-6) mRNA, a cytokine that is involved in angiogenesis and tumor progression process, increasing proliferation and invasion of the tumor [16, 18]. Furthermore, Shang et al. also reported that positive *β*2-AR immunoexpression in oral cancer is significantly correlated with age, tumor size, clinical stage, and cervical lymph node metastasis in OSCC patients, suggesting a role of *β*2-AR in the metastasis of oral cancer [16]. In contrast, our group, in a retrospective clinical study, showed that patients with OSCC, who exhibited strong *β*2-AR expression by malignant epithelial cells, presented higher survival rates compared with weak/negative *β*2-AR expression [21].

Although *β*2-AR is present in normal oral tissues, the exact differences between *β*2-AR expression levels in normal oral epithelium and oral cancer are not so clear [3, 16, 18, 21]; for example, *β*2-AR was highly expressed in OSCC tissue when compared to adjacent normal oral mucosa [16]. In turn, Bernabé et al. showed no significant differences between *β*2-AR mRNA levels in normal oral mucosa and OSCC specimens [18].

In order to contribute with recent studies and investigate the role of *β*2-AR in OSCC, the present study aimed to analyze the expression of *β*2-AR in the tumor-free surgical margin and at the invasive front of a large sample of OSCC to verify if *β*2-AR expression is correlated with tumor-free surgical margins and the invasive front of OSCCs and to explore associations between *β*2-AR expression levels and clinicopathological features.

## 2. Patients and Methods

### 2.1. Patients and Tumor Samples

The present retrospective study was based on the analysis of 62 patients previously studied by Bravo-Calderón et al. [21]. All patients were submitted to surgical treatment for primary oral squamous cell carcinoma at the Head and Neck Surgery and Otorhinolaryngology Department of the A.C. Camargo Cancer Center, São Paulo, Brazil, from 1970 to 2000. The inclusion criteria were (i) primary OSCC located in the oral tongue, floor of the mouth, retromolar area or inferior gingiva confirmed by biopsy; (ii) patients submitted to surgery as the initial treatment followed or not by radiotherapy; (iii) clinical stages II, III, and IV; and (iv) tumor and morphologically normal/nondysplastic tumor-free surgical margin tissues available for microscopic analysis. Patients with other simultaneous primary tumors, or with distant metastases at the time of admission, undergoing preoperative chemotherapy and/or radiotherapy were not included. Clinical data of the patients were collected from the hospital records and included age, gender, ethnic group, tobacco and alcohol consumption, tumor location, and disease stage according to the TNM system of the International Union Against Cancer (UICC) [26], and treatment (surgery, postoperative adjuvant radiotherapy) and clinical follow-up (recurrence, cervical lymph node metastasis, distant metastasis, or occurrence of second primary tumor). The present study was approved by the Research Ethics Committee of the A.C. Camargo Cancer Center, São Paulo, Brazil (#1385/10).

A formalin-fixed 3**** *μ*m thick section of tumor tissue was selected and paraffin-embedded for hematoxylin and eosin staining and immunohistochemistry analysis of *β*2-AR. Three observers (Denise Tostes Oliveira, Gustavo Amaral Lauand, Diego Mauricio Bravo-Calderón) analyzed the tumor sections without knowledge of the clinical data. Histopathological malignancy grade of OSCC was determined according to the Bryne et al. system [27]. Tumor infiltration to adjacent structures, vascular embolization, and lymph node metastases (pN+) were also reported.

### 2.2. Beta-2 Adrenergic Receptor Expression in the Tumor-Free Surgical Margin of Oral Squamous Cell Carcinoma

Sections of the 62 OSCC specimens were deparaffinized in xylene and hydrated using graded alcohol/water baths. Antigen retrieval was performed using 10** **mM citrate buffer (pH 6.0) in a domestic pressure cooker (Nigro, model Eterna 4.5 L, Araraquara, SP, Brazil) for 4** **min, and then the endogenous peroxidase activity was blocked by incubation in 3% H_2_O_2_ for 30** **min. Tumor sections were incubated for 18 hours at 4°C in a humid chamber with the anti-beta-2 adrenergic receptor primary antibody (Santa Cruz Biotechnology, sc-9042, Santa Cruz, CA, USA) and diluted 1 : 50 in phosphate buffered saline (PBS) with bovine serum albumin solution (Sigma, A9647, St. Louis, MO, USA) to block nonspecific reactions. Next, the tumor sections were sequentially incubated with Post Primary Block (Novocastra, NovoLink Max Polymer, RE7260-K, Newcastle Upon Tyne, UK) for 30** **min, followed by incubation with the Polymer from the same kit. The antigen-antibody reactions were revealed using 3.3′ diaminobenzidine tetrahydrochloride (DAB/Sigma, D-5637, St. Louis, MO, USA) for 5** **min in the dark. Tumor sections were counterstained with Harris hematoxylin before being dehydrated and prepared with a cover slip. The vascular smooth muscle within the sections served as the positive internal control. For a negative control, the primary antibody was omitted during the immunohistochemical staining. All the OSCC specimens were immunostained in a single session.

Ten microscopic fields of the tumor-free margins were captured using 400x magnification to analyze the immunohistochemical expression of *β*2-AR. Images were obtained digitally with a camera (Axiocam MRc, ZEISS, Jena, Germany) attached to a light microscope (Axioskop 2 Plus, ZEISS, Jena, Germany) and saved in a computer program system (Axiovision 4.6, ZEISS, Jena, Germany). The immunohistochemical expression of *β*2-AR was evaluated as previously described [21]. Briefly, the ImageJ software (Java-based image processing and analysis program of public-domain developed by Wayne Rasband NIH, Bethesda, MD, USA) was used to segment the representative tumor images. Variations in the brown intensity of the malignant cells positive for *β*2-AR expression were categorized according to the following RGB channel value ranges:

 R (red) channel was from 90 to 194;
 G (green) channel was from 50 to 140;
 B (blue) channel was from 45 to 147;
 the R value should be greater than the B value;
 the G value should be greater than the B value.

Ten images captured from the surgical margins of each OSCC were automatically segmented using the MATLAB computing language-based software according to the criteria listed above. This software measures, based on the number of pixels, each segmented area (determining the *β*2-AR immunopositive regions). After performing this computer-assisted immunohistochemistry analysis, the average of the *β*2-AR expression levels in ten surgical margin images was calculated. Next, the averages of 62 OSCC specimens, previously obtained by Bravo-Calderón et al. [21], were placed in ascending order and the median was established as the cut-off point to classify the specimens as exhibiting weak/negative (averages 0.62 to 23.86) or strong (averages 25.99 to 79.63) *β*2-AR expression. This measurement of *β*2-AR expression by epithelial cells of surgical margins was then subjectively confirmed by three investigators (Denise Tostes Oliveira, Gustavo Amaral Lauand, and Diego Mauricio Bravo-Calderón) without knowledge of the histopathological features and patient clinical status. In case of discordance between these analyzers, the criterion of the subjective evaluation was retained because this assessment involved the entire specimen.

In addition, knowing that most antigens are influenced adversely by formalin fixation [28], a confirmation of *β*2-AR expression pattern was conducted through immunohistochemical staining of 19 additional frozen sections of OSCC obtained from the Anatomical Pathology Service, Clinical Hospital University of Santiago, Santiago de Compostela, Spain.

### 2.3. Statistical Analysis

All statistical analysis was performed using the SPSS 13.0 for windows software (SPSS Inc., Chicago, IL, USA). Correlation between *β*2-AR expressions in the tumor-free surgical margins and at the invasive front of OSCCs was verified by Pearson's correlation coefficient test. The association between the *β*2-AR expression and clinicopathological variables was analyzed by the chi-square or Fisher's exact tests. For all tests, *p* ≤ 0.05 was considered to represent a statistically significant result.

## 3. Results

Immunohistochemical analysis of the 62 OSCC specimens revealed a positive expression of *β*2-AR in parakeratinized stratified squamous epithelium of all tumor-free surgical margins, and 50 (80.6%) demonstrated strong expression of *β*2-AR levels. *β*2-AR immunostaining was detected at the cytoplasm and at the plasma membrane of normal epithelial cells in tumor-free surgical margins. Similarly, the same pattern of *β*2-AR expression (cytoplasmic and membranous) was identified in malignant epithelial cells at the invasive front of tumors.

**Figure 1:**
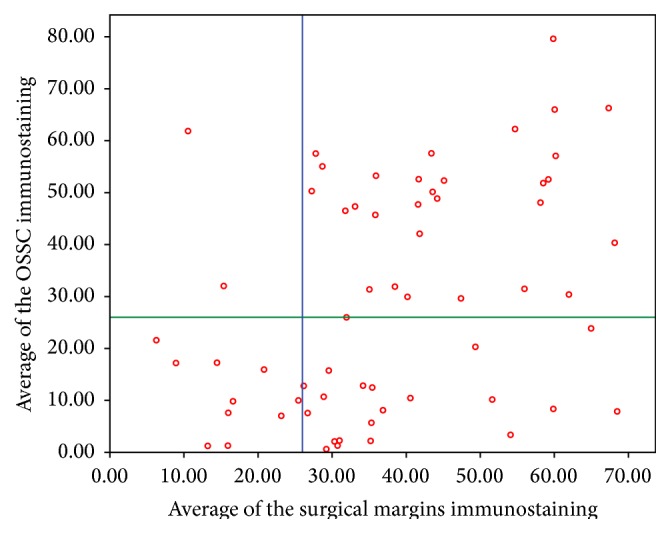
Correlation of the averages of *β*2-AR immunoexpression in OSCC specimens. The lines correspond to the cut-off point that classifies the invasive tumor fronts (green line) and the surgical margins (blue line) as exhibiting weak/negative (averages 0.62 to 23.86) or moderate/strong (averages 25.99 to 79.63) *β*2-AR expression.

Interestingly, Pearson's correlation coefficient test detected a statistically significant and positive correlation between the *β*2-AR expression levels in tumor-free surgical margins and at the invasive front of tumors (*r* = 0.383; *p* = 0.002) (Figure 1). Effectively, most (64.5%) of the 62 OSCC specimens had an increase of *β*2-AR expression levels in the tumor-free surgical margins, and it was accompanied by an increase of the expression levels at the invasive front. In other words, those specimens with weak *β*2-AR expression in the tumor-free surgical margin also exhibited weak/negative immunostaining of this protein in their respective invasive tumor front; and similarly, when the immunoexpression of *β*2-AR was strong in normal oral epithelial cells, at the tumor-free surgical margin, the expression levels of this protein in malignant epithelial cells at the invasive front of the tumor were also high (Figure 1).

**Figure 2:**
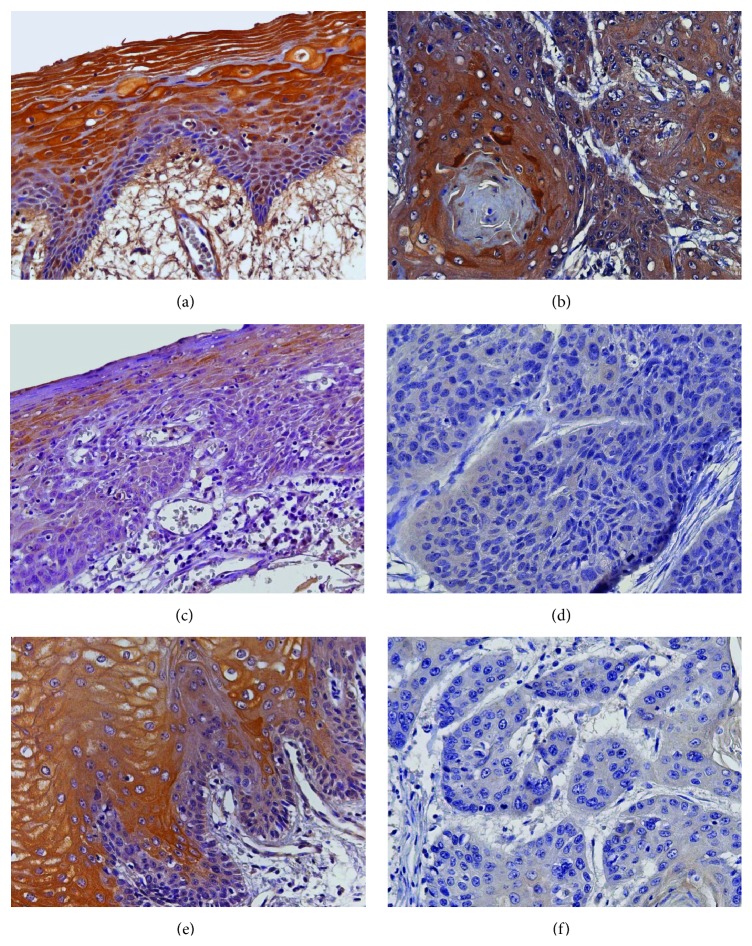
Expression pattern of *β*2-AR in OSCC specimens. (a and b) OSCC with strong expression of *β*2-AR in both surgical margin and invasive front of tumor. (c and d) OSCC with weak expression of *β*2-AR in both surgical margin and invasive front of tumor. (e and f) OSCC with reduced expression of *β*2-AR at the invasive front of the tumor when compared to surgical margin. Note the membranous and/or cytoplasmic expression pattern of *β*2-AR in immunostained cells (a, b, c, d, e, and f: immunohistochemistry *β*2-AR, original magnification ×400).

On the other hand, 22 (35.5%) OSCC samples presented no correlation between *β*2-AR levels in the tumor-free margins and at the invasive front of tumors, as shown in Figure 1. In 20 of these noncorrelated cases (32.25% of the total sample), the invasive front of tumor presented a decrease of *β*2-AR expression levels when compared to their respective tumor-free surgical margins (Figure 1). On the other hand, the pattern of *β*2-AR expression found in the tumor-free surgical margin and at the invasive tumor front of OSCC is illustrated in Figure 2.

An additional analysis was performed to confirm our findings in formalin-fixed, paraffin-embedded tissue material by the evaluation of 19 new OSCC frozen specimens. *β*2-AR expression, in this sample, was verified in most OSCC specimens, being positively immunoexpressed in 87.5% of the tumor-free surgical margins and in 55.5% of the front of invasion. In the basal and corneum layers of the surgical margins or in the keratin pearls of oral cancers, no immunoexpression of *β*2-AR was detected. Furthermore, the cytoplasmic and membranous expressions of *β*2-AR were confirmed in oral epithelial cells adjacent to the tumor, as well as in the malignant epithelial cells of OSCC.

### 3.1. Associations between *β*2-AR Expression and Clinicopathological Variables

To determine the possible clinical significance of *β*2-AR expression in oral cancer and the associations between this protein expression and the clinicopathological features of OSCC patients, chi-square or Fischer's exact tests were performed.

No statistically significant associations were found regarding immunohistochemical expression of *β*2-AR in the tumor-free surgical margins and clinical variables evaluated (Table 1). On the other hand, *β*2-AR immunoexpression at the invasive tumor front was statistically associated with alcohol consumption (*p* = 0.038), simultaneous consumption of alcohol, and tobacco (*p* = 0.010) and with the clinical T stage (*p* = 0.014), as shown in Table 1. Most OSCC patients with weak/negative expression of *β*2-AR at the invasive front of tumor exhibited alcohol consumption or alcohol and tobacco consumption. In addition, strong *β*2-AR expression by malignant epithelial cells was more frequently detected in patients with early clinical stage of T1/T2.

Regardless of the area that was considered for analysis, either the tumor-free surgical margin or the invasive tumor front, no statistically significant association between *β*2-AR expression and the histopathological characteristics was shown, including grade of malignancy, lymph nodes involvement (pN+), vascular embolization and perineural invasion, and muscular or bone infiltration (Table 2).

## 4. Discussion

Although several studies have described the functional localization of *β*2-AR in a wide variety of cells including those of the tumor microenvironment, the expression pattern of this receptor in normal oral epithelium and in malignant epithelial cells is not well defined yet [1–3, 6, 8–24]. Our immunohistochemical analysis of a large cohort of OSCC specimens has shown a cytomembranous expression of *β*2-AR in normal oral epithelial cells of all tumor-free surgical margins, except for the corneum and basal layers (Figure 2). These findings corroborate previous reports that identified *β*2-AR as the main adrenergic receptor subtype in cultured human oral keratinocytes and those in which a RT-PCR assay verified the expression of *β*2-AR mRNA in 14 of 15 specimens of normal oral mucosa [3, 18]. Collectively, data obtained by us and by mentioned reports [3, 18] suggest that *β*2-AR is constitutively expressed in normal oral epithelium.

Concerning OSCC, similarly to normal epithelium, the invasive front of tumor is considered as the most progressed region by Piffkò et al. [29], and approximately three to six tumor cell layers or detached tumor cell groups were positively immunostained for *β*2-AR at the cytoplasm and plasma membranes of malignant epithelial cells, except for the keratin pearls that were negative (Figure 2). In addition, the present study is the first to demonstrate that the expression of *β*2-AR in surgical margins is positively correlated with the invasive front of tumor expression levels (*r* = 0.383; *p* = 0.002) (Figure 1). Despite methodological differences, our findings corroborate the observations proposed by Bernabé et al. [18] which reported no expressive difference in *β*2-AR mRNA expression in not matched specimens of normal mucosa and OSCC [18]. Contradictorily, Shang et al. [16] reported that *β*2-AR immunoexpression is higher in OSCC tissue than in the adjacent normal oral mucosa. However, comparing with our present results, it is impracticable because the aforementioned analysis was carried out in a small number of surgical margins. Besides, evaluations through correlation tests were not performed by Shang et al. [16], and according to the authors, their findings of *β*2-AR immunostaining in surgical margins might be caused by the diversity of experiments [16]. Moreover, the accuracy of our findings determined in formalin-fixed, paraffin-embedded tissues was validated positively by the analysis of the OSCC frozen specimens.

Biological evidence has demonstrated that chronic psychological stress through catecholamine-induced activation of *β*2-AR can influence the progression of a wide variety of malignant tumors [1, 2, 6–25]. However, the effects of stress related hormones can be stimulatory or inhibitory, depending on the type of hormone and the tumor type [25]. In this context, the role of *β*2-AR in the progression of oral cancer is not well established. Thus, the scientific literature has demonstrated that *β*2-AR immunoexpression by malignant cells is significantly correlated with age, tumor size, clinical stage, and cervical lymph node metastasis in OSCC patients [16]. Nevertheless, the present clinical study revealed that patients clinically classified as T3 or T4 exhibited a higher frequency of weak/negative *β*2-AR expression at the invasive tumor front (Table 1). In addition, considering that *β*2-AR was presented in all tumor-free surgical margins, it was expected that no statistically significant associations would be found regarding immunohistochemical expression in the normal oral epithelial cells and clinicopathological variables evaluated (Tables 1 and 2).

On the other hand, the evidence that, in certain OSCCs, *β*2-AR expression by tumoral cells may decrease (Figure 1), as previously shown in patients with oral cancer and weak/negative *β*2-AR expression and low survival rates, compared with strong *β*2-AR expression [21], reinforces previous findings obtained by Yu et al. [10]. Yu et al. demonstrated that genetic silencing of *β*2-AR increases cell migration and invasion in normal prostate cells and that weak expression of this protein was associated with metastases and worst survival rates in prostate cancer patients. Considering the present results, other studies with *β*2-AR expression should be performed to further elucidate the role of this receptor. In vitro analyses are suggested with the stimulatory and inhibitory factors for *β*2-AR.

## 5. Conclusions

Thereby, in light of these results we can conclude that although the present study reinforces that *β*2-AR is constitutive in normal oral epithelial cells and is positively correlated with the expression levels of *β*2-AR by OSCC cells, further clinical, cellular, and animal studies are needed to elucidate the role of *β*2-AR in oral cancer, specially, in relation to the importance of its decrease on tumor progression.

## Figures and Tables

**Table 1:** Association between clinical parameters and *β*2-AR expression in 62 patients with oral squamous cell carcinoma.

Variable		Beta-2 adrenergic receptor					
		Tumor-free margin			Invasive tumor front		
		Weak (*N* = 12)	Moderate/strong (*N* = 50)	*p*	Weak/negative (*N* = 36)	Moderate/strong (*N* = 26)	*p*
		*N* (%)	*N* (%)		*N* (%)	*N* (%)	
Gender	Male	8 (66.7)	44 (88)	0.091	31 (86.1)	21 (80.8)	0.729
	Female	4 (33.3)	6 (12)		5 (13.9)	5 (19.2)	

Ethnic group	White	10 (83.3)	45 (90)	0.612	33 (91.7)	22 (84.6)	0.439
	Not white	2 (16.7)	5 (10)		3 (8.3)	4 (15.4)	

Age	≤58 years	8 (66.7)	25 (50)	0.299	22 (61.1)	11 (42.3)	0.143
	>58 years	4 (33.3)	25 (50)		14 (38.9)	15 (57.7)	

Tobacco^#^	Yes	11 (100)	42 (93.3)	0.999	31 (100)	22 (88)	0.083
	No	0 (0)	3 (6.7)		0 (0)	3 (12)	

Alcohol^#^	Yes	10 (90.9)	32 (69.6)	0.256	27 (84.4)	15 (60)	**0.038^*∗*^**
	No	1 (9.1)	14 (30.4)		5 (15.6)	10 (40)	

Tobacco + alcohol^#^	Yes	10 (90.9)	29 (64.4)	0.144	26 (83.9)	13 (52)	**0.010^*∗*^**
	No	1 (9.1)	16 (35.6)		5 (16.1)	12 (48)	

T stage	T1/T2	6 (50)	28 (56)	0.708	15 (41.7)	19 (73.1)	**0.014^*∗*^**
	T3/T4	6 (50)	22 (44)		21 (58.3)	7 (26.9)	

N stage	N+	6 (50)	24 (48)	0.901	18 (50)	12 (46.2)	0.765
	N0	6 (50)	26 (52)		18 (50)	14 (53.8)	

Clinical stage	II	3 (25)	16 (32)	0.74	9 (25)	10 (38.5)	0.257
	III/IV	9 (75)	34 (68)		27 (75)	16 (61.5)	

Recurrence^##^	Yes	6 (50)	20 (40)	0.528	17 (47.2)	9 (34.6)	0.321
	No	6 (50)	30 (60)		19 (52.8)	17 (65.4)	

Metastases	Yes	0 (0)	1 (2)	0.999	0 (0)	1 (3.8)	0.419
	No	12 (100)	49 (98)		36 (100)	25 (96.2)	

Second primary tumor	Yes	1 (8.3)	8 (16)	0.675	5 (13.9)	4 (15.4)	0.999
	No	11 (91.7)	42 (84)		31 (86.1)	22 (84.6)	

*N*: number of cases; *p*: *p* value obtained by chi-square test or Fisher's exact test; ^#^excluding patients with lost records; ^##^local and/or regional recurrence; ^*∗*^statistically significant result.

**Table 2:** Association between histopathological parameters and *β*2-AR expression in 62 patients with oral squamous cell carcinoma.

		Beta-2 adrenergic receptor					
Variable		Tumor-free margin			Invasive tumor front		
		Weak (*N* = 12)	Moderate/strong (*N* = 50)	*p*	Weak/negative (*N* = 36)	Moderate/strong (*N* = 26)	*p*
		*N* (%)	*N* (%)		*N* (%)	*N* (%)	
Malignancy grading	M. diff.	10 (83.3)	44 (88)	0.645	31 (86.1)	23 (88.5)	0.999
	L. diff.	2 (16.7)	6 (12)		5 (13.9)	3 (11.5)	

Vascular embolization	Yes	9 (75)	26 (52)	0.149	23 (63.9)	12 (46.2)	0.165
	No	3 (25)	24 (48)		13 (36.1)	14 (53.8)	

Perineural infiltration	Yes	9 (75)	36 (72)	0.999	24 (66.7)	21 (80.8)	0.219
	No	3 (25)	14 (28)		12 (33.3)	5 (19.2)	

Muscular infiltration	Yes	11 (91.7)	43 (86)	0.999	31 (86.1)	23 (88.5)	0.999
	No	1 (8.3)	7 (14)		5 (13.9)	3 (11.5)	

Bone infiltration^#^	Yes	1 (8.3)	5 (10.2)	0.999	5 (14.3)	1 (3.8)	0.227
	No	11 (91.7)	44 (89.8)		30 (85.7)	25 (96.2)	

Lymph node involvement	*p*N+	4 (33.3)	26 (52)	0.245	18 (50)	12 (46.2)	0.765
	*p*N0	8 (66.7)	24 (48)		18 (50)	14 (53.8)	

*N*: number of cases; *p*: *p* value obtained by chi-square test or Fisher's exact test; M. diff.: more differentiated tumor; L. diff.: less differentiated tumor; ^#^excluding patients with lost records.

## Abbreviations

*β*2-AR: Beta-2 adrenergic receptor
OSCC: Oral squamous cell carcinoma.
